# Exploration of phyllosphere microbiomes in wheat varieties with differing aphid resistance

**DOI:** 10.1186/s40793-023-00534-5

**Published:** 2023-10-24

**Authors:** Xinan Li, Chao Wang, Xun Zhu, Vardis Ntoukakis, Tomislav Cernava, Decai Jin

**Affiliations:** 1 State Key Laboratory for Biology of Plant Diseases and Insect Pests, Institute of Plant Protection, Chinese Academy of Agricultural Sciences, 100193 Beijing, China; 2 Henan Engineering Research Center of Biological Pesticide & Fertilizer Development and Synergistic Application, School of Resource and Environmental Sciences, Henan Institute of Science and Technology, 453003 Xinxiang, China; 3 School of Life Sciences, University of Warwick, CV4 7AL Coventry, UK; 4 Institute of Environmental Biotechnology, Graz University of Technology, Petersgasse 12, 8010 Graz, Austria; 5 School of Biological Sciences, Faculty of Environmental and Life Sciences, University of Southampton, SO17 1BJ Southampton, UK; 6 Key Laboratory of Environmental Biotechnology, Research Center for Eco-Environmental Sciences, Chinese Academy of Sciences, 100085 Beijing, China

**Keywords:** Bacterial community, Endophytes, Plant-microbe interactions, Phyllosphere, Wheat aphids

## Abstract

**Background:**

Leaf-associated microbes play an important role in plant development and response to exogenous stress. Insect herbivores are known to alter the phyllosphere microbiome. However, whether the host plant’s defense against insects is related to the phyllosphere microbiome remains mostly elusive. Here, we investigated bacterial communities in the phyllosphere and endosphere of eight wheat cultivars with differing aphid resistance, grown in the same farmland.

**Results:**

The bacterial community in both the phyllosphere and endosphere showed significant differences among most wheat cultivars. The phyllosphere was connected to more complex and stable microbial networks than the endosphere in most wheat cultivars. Moreover, the genera *Pantoea*, *Massilia*, and *Pseudomonas* were found to play a major role in shaping the microbial community in the wheat phyllosphere. Additionally, wheat plants showed phenotype-specific associations with the genera *Massilia* and *Pseudomonas*. The abundance of the genus *Exiguobacterium* in the phyllosphere exhibited a significant negative correlation with the aphid hazard grade in the wheat plants.

**Conclusion:**

Communities of leaf-associated microbes in wheat plants were mainly driven by the host genotype. Members of the genus *Exiguobacterium* may have adverse effects on wheat aphids. Our findings provide new clues supporting the development of aphid control strategies based on phyllosphere microbiome engineering.

**Supplementary Information:**

The online version contains supplementary material available at 10.1186/s40793-023-00534-5.

## Background

Insect herbivores are a pervasive threat to plants in agricultural and natural settings alike [1]. Herbivores can induce various plant defense mechanisms, which can alter the sensitivity of plants to insects and microbial attacks [2–4]. The feeding of herbivores also affects the colonization and growth of plant-associated microorganisms in the host [5]. It is estimated that leaves comprise a major proportion of the Earth’s total biomass, making it an important habitat for microbes [6]. Leaves are colonized by a high diversity of epiphytic and endophytic microbes [7]. A previous study showed that insect herbivory may drive the epidemiology of plant-infecting bacteria as well as the structure of the native plant microbiome by causing changes in the fitness of bacteria within the host at multiple phylogenetic and spatial scales [5].

Phyllosphere microbial communities are not randomly assembled and distinct bacteria can be enriched therein [8, 9]. Phenotypic traits of different genotypes in the same plant species may be significantly different (e.g., leaf length, leaf width, and level of resistance to pests and diseases). Some studies have shown that the composition of the plant microbiome is highly specific in different genotypes [10], while other studies have shown that the host genotype has a weak effect on plant-associated microorganisms [11]. Additionally, plant-associated microbial communities are affected by various abiotic factors (e.g., rainfall, temperature, UV radiation, etc.), biotic factors (e.g., pollinators, microbial interactions, etc.), and anthropogenic activities such as agricultural practices [12, 13].

Leaves provide a specific environment for microbial colonization, where the microbiota plays crucial roles in host performance and resilience to environmental perturbations [14–16]. For instance, there are many beneficial microbes in the plant phyllosphere, which can directly promote plant growth by improving nutrient acquisition or hormone stimulation, or indirectly affect plant health by inhibiting the growth of plant pathogens through competition and antagonism [16–18]. Moreover, phyllosphere microbes can degrade compounds harmful to plants, humans, or the environment, such as phenols [19], hydroquinones [20], and polyaromatic hydrocarbons [21]. Additionally, plant-associated microbes can influence flowering time [4], improve resistance to salinity and drought stress [22], and influence other host physiological traits. Therefore, a detailed understanding the mechanism of of plant microbiome assembly, function, and microbial co-occurrence networks is essential for developing microbial-based solutions for sustainable crop production systems.

Wheat (*Triticum aestivum*) is a commonly cultivated crop in China, and is one of the main cereal plants in many other countries. Currently, wheat production is increasingly threatened by aphids (i.e., *Sitobion avenae* and *Rhopalosiphum padi*), resulting in 10-40% yield losses annually in China [23, 24]. Previous research has shown that the bacterial genera *Acinetobacter*, *Microbacterium*, *Psychrobacter*, *Bacillus*, *Proteus*, *Streptomyces*, *Pseudomonas*, and *Kineococcus* were prevalent in the phyllosphere of different wheat varieties in Pakistan, and connected with plant growth promotion in high-yield varieties [25]. Moreover,* Pseudomonads* in wheat leaves play an important role due to their antagonistic effects towards the fungal pathogens *Fusarium* and *Alternaria*, and contribute to natural plant protection [26]. However, the diversity and structure of leaf-related bacterial communities in different wheat cultivars, and whether the number of aphid occurrences is related to these bacterial communities is still unexplored.

In this study, the composition of bacterial communities was investigated using amplicon sequencing of 16S rRNA gene fragments in the phyllosphere and endosphere of eight wheat cultivars, which were grown in the same farmland. These cultivars also exhibit varying levels of resistance to aphids. We explored differences in leaf-associated bacterial communities among the eight cultivars, identified core species affecting leaf-associated microbiome, and determined the relationship between aphid occurrence and microorganisms. This study has important implications for uncovering interactions, functions, and mutualistic relationships between plants and their associated microbiota, as well as for developing new aphid control strategies.

## Methods

### Sample collection

All wheat leaf samples were collected in 2021 from an experimental field in Xinxiang, Henan, China (113°48′18.08″E, 35°09′12.72″N). The eight wheat cultivars with different aphid resistance were selected based on previous work. Information related to leaf length, leaf width, and aphid hazard grade (AG) of the different cultivars is listed in Table 1 and Table S1. Each cultivar was planted in 10 rows in a small plot with an area of 2 × 2 m, and one meter apart from other cultivars. All plants were managed in the same way (including fertilization and irrigation). Wheat leaf samples were collected at the filling stage without any visible signs of plant diseases. One or two leaves from the upper part of plants in each row were randomly collected, 10 leaves were used as a sample with eight biological replicates, and transported to the laboratory with cooling packs.

**Table 1:** Information on leaf length, leaf width and aphid hazard grade (AG) of different wheat cultivars

Code	Cultivar	Leaf length ± SE/cm	Leaf width ± SE/cm	AG ± SE
HR16	Heng-r16-5152	18.71 ± 0.70	2.12 ± 0.06	3.28 ± 0.15
L112	Luo-11238-147-41	18.77 ± 0.43	2.15 ± 0.06	1.34 ± 0.12
L01	Lan-01-368	22.71 ± 0.48	2.16 ± 0.04	2.34 ± 0.10
H05	Han-05-5093	23.42 ± 0.72	2.20 ± 0.06	1.25 ± 0.07
BL40	BL4071	26.45 ± 1.01	2.24 ± 0.07	2.31 ± 0.09
K13	Ke-13-487	17.98 ± 0.20	2.10 ± 0.04	2.00 ± 0.13
M15	Mian-15Z30	23.11 ± 0.41	2.27 ± 0.05	2.03 ± 0.11
XD17	Xindong-17	19.14 ± 0.66	2.12 ± 0.05	2.50 ± 0.16

Values are the average of 8 biological replicates for leaf length and width; SE, standard error; AG, aphid hazard grade, ten rows of each cultivar were investigated and repeated three times; AG is divided into five grades: 0, no aphids in whole plant; 1, 1–10 aphids in whole plant; 2, 10–20 aphids in whole plant; 3, 21–50 aphids in whole plant; 4, more than 50 aphids in whole plant

Enrichment of microorganisms was conducted following the methodology of a previous study [27]. Briefly, wheat leaves were immersed in sterile PBS buffer (pH 7.0, 0.02 mM, 0.1% Tween 80), oscillated on a 30 °C thermostatic oscillator for 30 min, and then sonicated for 10 min. For phyllosphere microorganisms, the suspension was passed through a 0.22 μm membrane using a vacuum pump, and then the membrane containing microorganisms was stored at 4 °C until DNA extraction. For endosphere microbes, after the collection of phyllosphere microbes, leaf surfaces were sterilized with 75% ethanol for 3 min, 2.5% sodium hypochlorite (NaClO) for 5 min, and then rinsed five times with sterile water. The leaves were ground with a tissue grinder to extract total DNA from the samples.

### High-throughput sequencing

DNA Extraction, PCR Amplification, and high-throughput sequencing were performed based on previously described protocols [27]. Fragments of the 16S rRNA gene were amplified using primer pair (799 F: AACMGGATTAGATACCCKG, 1115R: AGGGTTGCGCTCGTTG) [28]. A 12-bp unique barcode was included in each primer pair to distinguish among sequenced samples. The sequencing was performed by Magigene Biotechnology Co., Ltd. (Guangzhou, China). A total of 128 samples was sequenced in this study (endosphere and phyllosphere, 8 cultivars, 8 replicates; 2 × 8 × 8 = 128).

### Data analysis

An online platform (http://mem.rcees.ac.cn:8080/) was used to analyze the raw data [29]. FLASH was used to combine forward and reverse sequence files into full-length sequences [30]. Reads containing ambiguous nucleotides (N) or average quality score of less than 20 were removed from the analysis. The sequences were then trimmed based on their length. Uparse tool and SILVA database 138.1 version were used to generate an operational taxonomic unit (OTU) table at 97% similarity level [31, 32], and to conduct taxonomic assignments [33]. OTU tables were randomly resampled to assess changes in sequencing depth. The sequencing data is publicly available at the NCBI Sequence Read Archive under accession no. PRJNA923742.

The alpha diversity and richness of microbial communities were evaluated by calculating the Shannon and Chao1 indexes, respectively. A principal coordinate analysis (PCoA) was performed based on a weighted Unifrac matrix for visualizing beta diversity. The differences in endosphere and phyllosphere bacterial communities of different wheat cultivars were evaluated using dissimilarity tests. Microbial community functions were predicted with Tax4Fun [34]. A Mantel test based on both Jaccard distances and Bray-Curtis was conducted to investigate the relationship between bacterial community structures and aphid hazard grade (AG) in different wheat cultivars.

Interaction networks were constructed to evaluate the interaction among different taxa of bacterial communities. The resampled OTU table of each wheat cultivar was used to construct individual networks using an online pipeline (http://ieg4.rccc.ou.edu/mena) [35]. Logarithmic data conversion was not implemented, and Spearman’s Rho was used for correlation calculation [36, 37]. Using the RMP method to construct the network, the thresholds (cut-off) for the phyllosphere and endosphere were 0.91 and 0.83, respectively. Network attributes such as mean path length, mean connectivity, and mean clustering coefficient (avgCC) for each dataset were calculated separately. Finally, the network was visualized using Cytoscape v3.3.0 [38]. The Zi-Pi threshold is based on the metabolic network approach described previously [39]. Briefly, we assigned all OTUs into four groups: peripherals (z_i_ ≤ 2.5; p_i_ ≤ 0.62), connectors (z_i_ ≤ 2.5; p_i_ > 0.62), module hubs (z_i_ > 2.5; p_i_ ≤ 0.62), and network hubs (z_i_ > 2.5; p_i_ > 0.62) [40]. Network hubs, module hubs, and connectors are keystone network topological features and are considered to play important roles in the stability and resistance of microbial communities; consequently, OTUs associated with these nodes were defined as keystone species [41].

IBM SPSS statistical software was used for statistical analysis. One-way ANOVA was used for testing significance, Tukey’s test was used for multiple comparisons, and Spearman’s correlation coefficient was used for correlation analysis.

## Results

### Diversity and structures of leaf-associated bacterial communities in different cultivars

A total of 6,563,333 and 7,047,320 high-quality bacterial sequences were retained in the wheat phyllosphere and endosphere bacterial community, respectively, after quality control. Following resampling for data normalization of 16S rRNA gene fragment reads, 37,333 and 23,683 reads were retained for wheat phyllosphere and endosphere bacterial communities, respectively. Bacterial OTUs of each wheat phyllosphere and endosphere were taxonomically classified, resulting in 233 to 923 and 189 to 1327 OTUs, respectively. Each rarefaction curve reached an asymptote at this sequencing depth (Figure S1), indicating sufficient sampling depth. OTU overlaps among the endosphere and phyllosphere bacterial communities were observed among the wheat cultivars (Figure S2); 209 and 462 OTUs were shared in the phyllosphere and endosphere of the eight wheat cultivars, respectively.

**Fig. 1:**
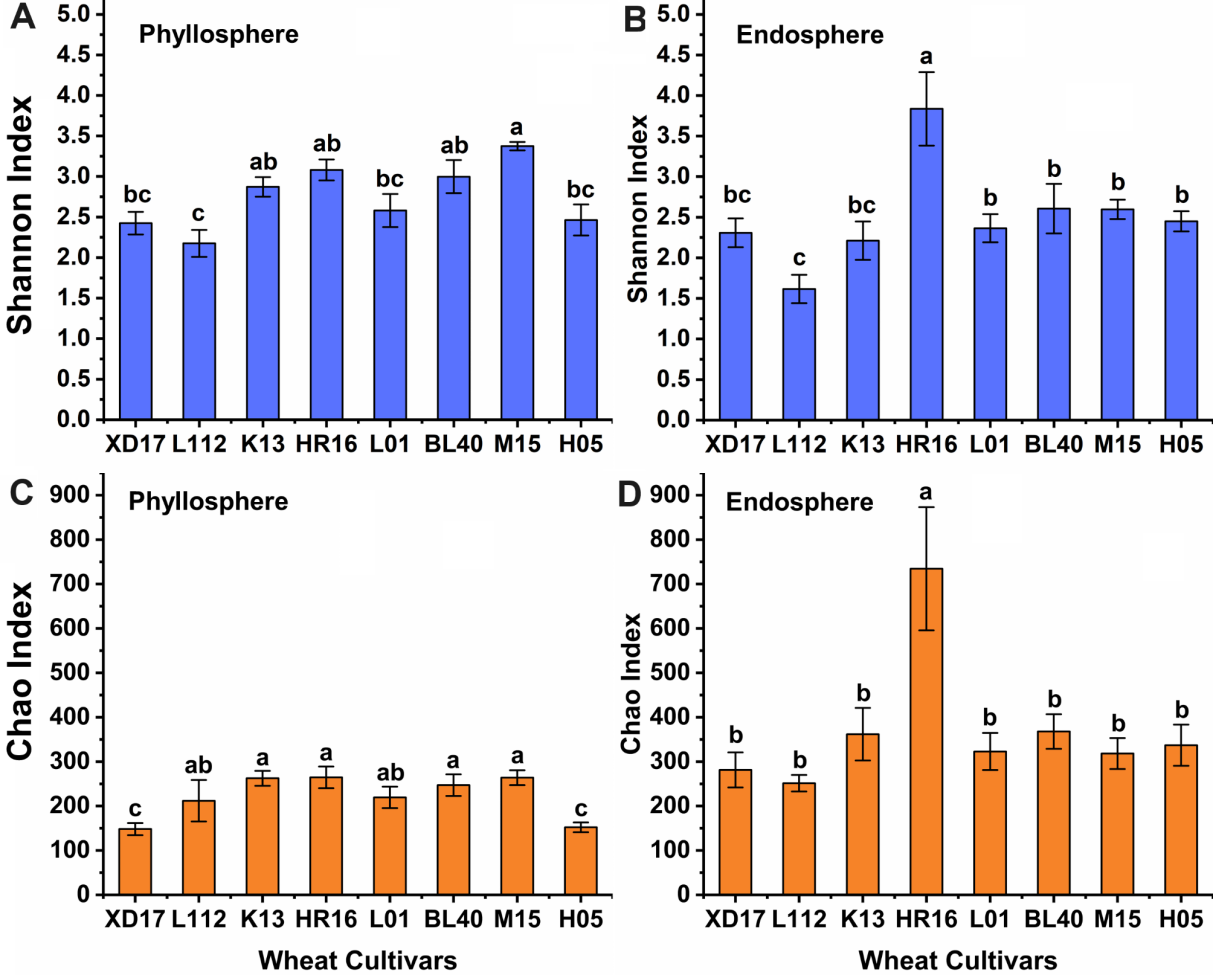
Shannon **(A, B)** and Chao1 index **(C, D)** of phyllosphere and endosphere bacterial communities of eight different wheat cultivars. Different letters indicate significant differences (*P* < 0.05)

The Shannon index was applied to analyze the alpha diversity of bacterial communities (Fig. 1A and B). For phyllosphere bacterial communities, the cultivar L112 had the lowest alpha diversity, while cultivar M15 had the highest alpha diversity. For endosphere bacterial communities, cultivars M15 and HR16 had the highest alpha diversity for phyllosphere and endosphere bacterial communities, respectively. The Chao1 index indicated differences in richness of bacterial species in different samples (Fig. 1C and D). For phyllosphere bacterial communities, the richness of K13, HR16, BL40, and M15 was significantly higher than that of cultivars XD17 and H05. For endosphere bacterial communities, the richness of cultivar HR16 was highest in all assessed cultivars.

**Fig. 2:**
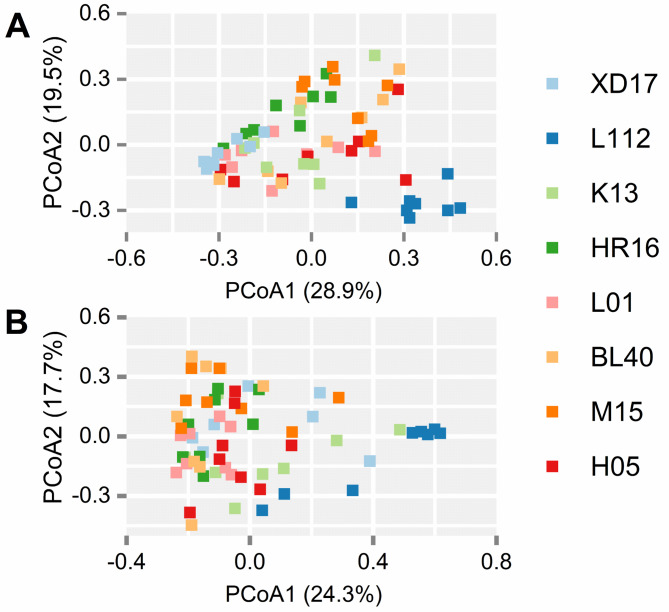
Principal coordinate analysis (PCoA) plots based on a weighted UniFrac matrix of bacterial communities in the phyllosphere **(A)** and endosphere **(B)** of eight wheat cultivars

A PCoA plot was used to visualize differences in bacterial community structures of different samples based on beta diversity (Fig. 2), and dissimilarity tests were conducted based on PERMANOVA (Table S2 and S3). The results indicated that the bacterial community in both the phyllosphere and endosphere showed significant differences among most wheat cultivars (*P* < 0.05).

### Leaf-associated bacterial community composition in different cultivars

**Fig. 3:**
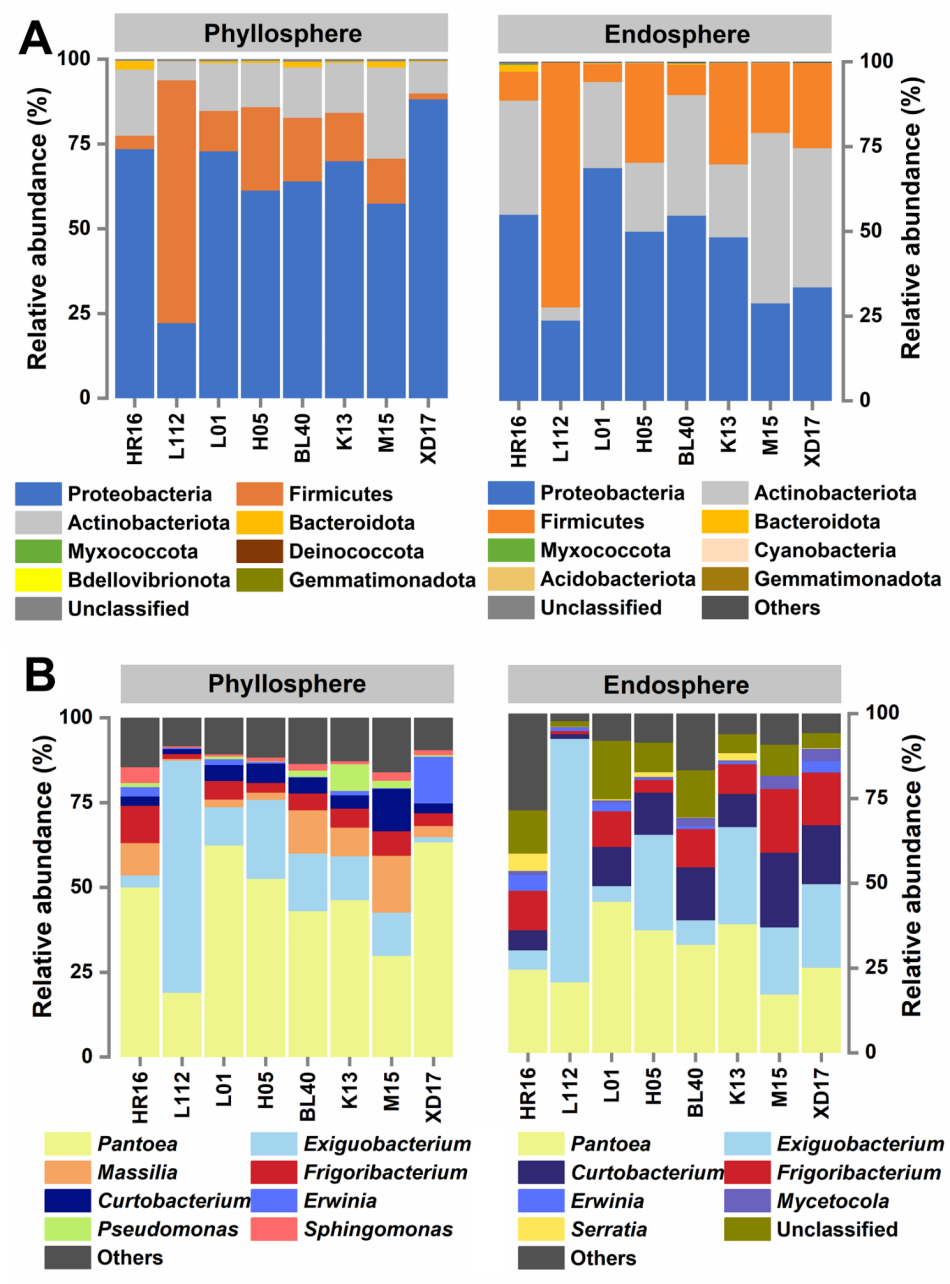
Bacterial community composition in the phyllosphere and endosphere at phylum **(A)** and genus **(B)** level. The fraction labeled with “other” represents the sum of all bacterial community members with a relative abundance < 2% in all cultivars

The bacterial community compositions of the wheat phyllosphere and endosphere at phylum level are shown in Fig. 3A. The phyllosphere bacterial communities were primarily composed of the phyla Proteobacteria (22.30–88.30%), Firmicutes (1.75–71.59%), Actinobacteria (5.63–26.86%), and Bacteroidota (0.33–2.59%). Among them, the average relative abundance of Proteobacteria was the highest in the phyllosphere of cultivar XD17 and the lowest in the phyllosphere of cultivar L112. The endosphere bacterial communities were primarily composed of the phyla Proteobacteria (22.82–68.79%), Firmicutes (5.31–72.21%), Actinobacteria (3.88–50.21%), and Bacteroidota (0.01–1.20%). Among them, the mean relative abundance of Proteobacteria in the endosphere of cultivar XD17 and L112 was respectively the highest and lowest. The mean relative abundance of Firmicutes was the highest in the phyllosphere and endosphere of cultivar L112, and Actinobacteria abundance was the highest in the endosphere of cultivar M15.

All samples were further analyzed regarding the composition of bacterial communities at genus level with a relative abundance threshold > 1% (Fig. 3B). The phyllosphere bacterial communities were primarily composed of the genera *Pantoea* (19.13–63.41%), *Exiguobacterium* (1.56-68.429%), *Massilia* (0.50-16.77%), *Frigoribacterium* (1.46–10.92%), *Curtobacterium* (1.52–12.47%), *Erwinia* (0.28–13.81%), *Pseudomonas* (0.03–7.89%), and *Sphingomonas* (0.46–4.65%). The endosphere bacterial communities were primarily composed of the genera *Pantoea* (17.27–44.63%), *Exiguobacterium* (4.70-71.76%), *Curtobacterium* (1.40–22.00%), *Frigoribacterium* (0.91–18.75%), *Erwinia* (0.14–4.66%), *Mycetocola* (0.29–3.80%), *Serratia* (0.02–5.14%), and various unclassified species (5.59–17.16%). Notably, the mean relative abundance of *Pantoea* was the highest in the endosphere of cultivar XD17, while *Exiguobacterium* abundance was the highest in the phyllosphere and endosphere of cultivar L112. Additionally, *Pantoea*, *Exiguobacterium*, *Frigoribacterium*, *Curtobacterium*, and *Erwinia* are shared bacterial genera in the phyllosphere and endosphere of the eight wheat cultivars.

### Identification of community modulators via microbial interaction networks

**Fig. 4:**
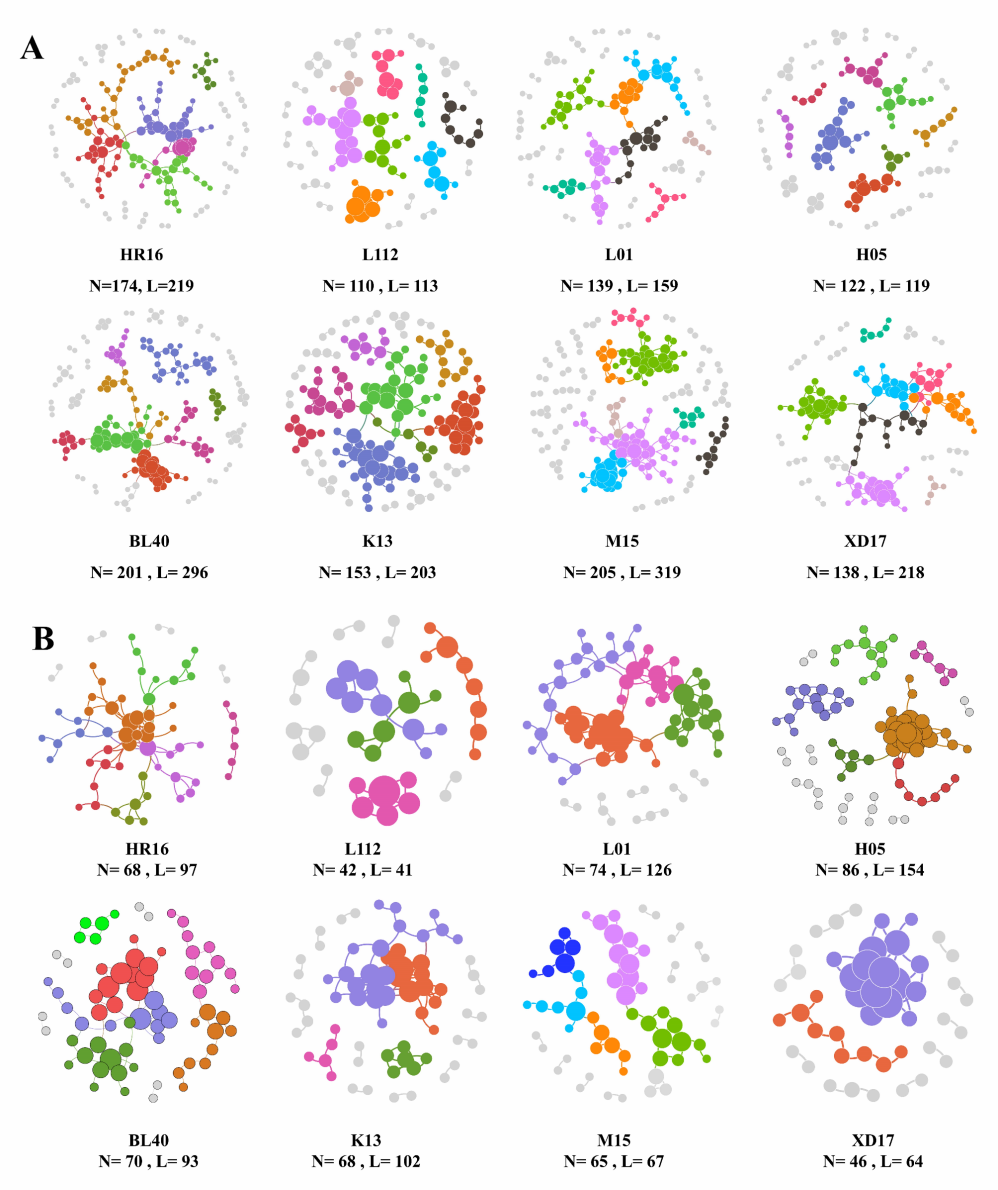
Network interactions in the phyllosphere **(A)** and endosphere** (B)** of eight wheat cultivars. Different colors indicate different modules

Different networks were constructed to infer intra-community interactions. The network and topological properties of endosphere and phyllosphere bacterial communities of the different wheat cultivars are shown in Fig. 4 and Table S4, respectively. The microbial interaction networks indicate that there are different nodes and links in the phyllosphere and endosphere of different wheat varieties, which were scale-free, not random, showed small-world properties, and could be divided into modules. These key topological properties qualified the constructed networks for further analysis. Additionally, the total links, nodes, and modules were significantly higher in the phyllosphere than in the endosphere of most wheat cultivars, indicating that the phyllosphere hosted more complex and stable networks, with closer and better-connected nodes.

**Fig. 5:**
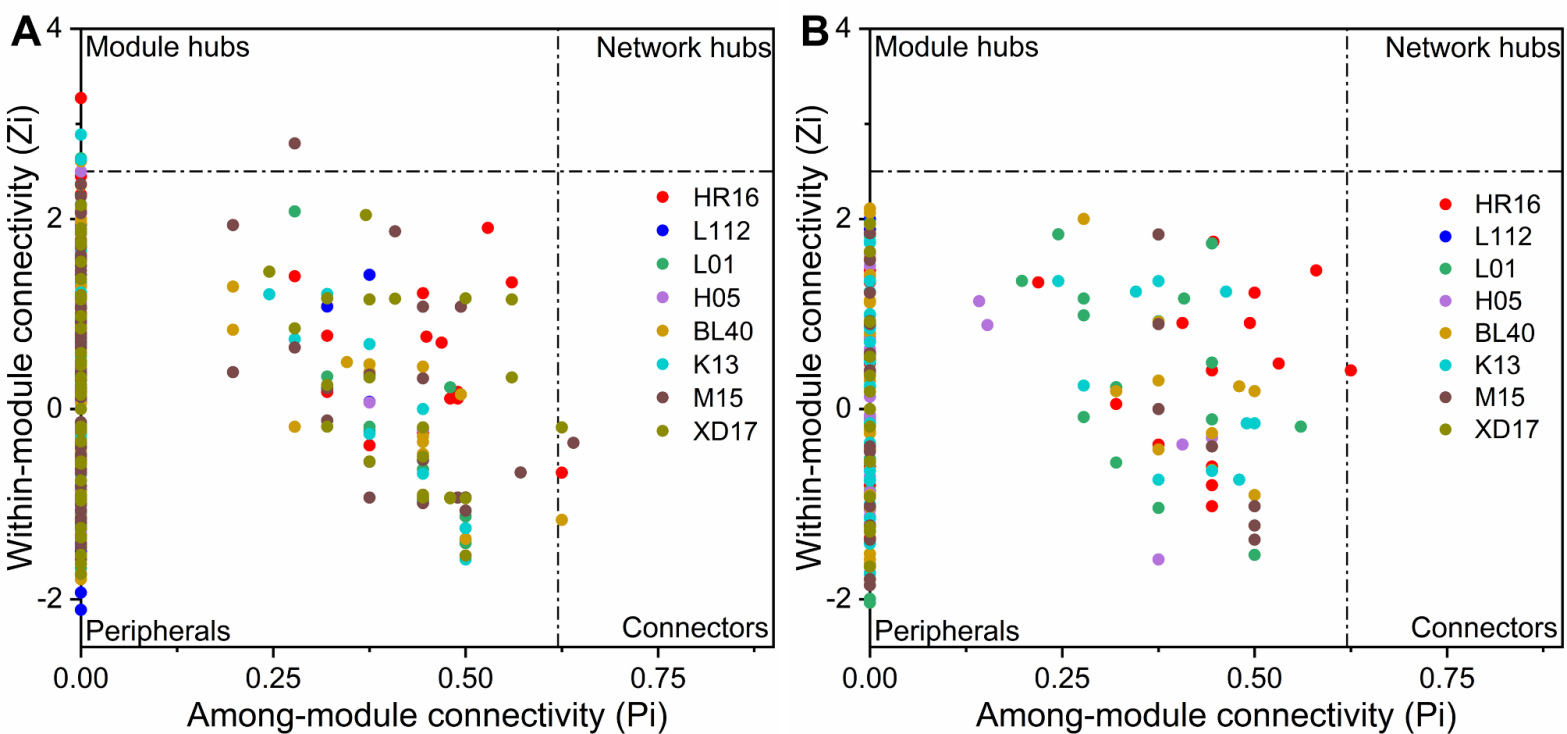
Z-P plots showing the distribution of OTUs based on their topological roles in the wheat phyllosphere **(A)** and endosphere **(B)**. Each dot represents an OTU. Threshold values of Zi and Pi for categorizing OTUs were 2.5 and 0.62, respectively

For the different wheat varieties, 113–319 links were identified in the phyllosphere, including 100–292 (88.50-99.54%) positive and 1–34 (0.46–11.50%) negative interactions, while 41–154 links were identified in the endosphere, including 36–144 (64.95–93.51%) positive and 5–34 (6.49–35.05%) negative interactions (Table S5). Z-P plot analyses were implemented to further define the roles of individual nodes within the networks (Fig. 5). For the Z-P plot analyses of the phyllosphere, most nodes were assigned to peripherals (99.20%). Six nodes were classified as module hubs in cultivars BL40, HR16, K13 (2 nodes), L01, and M05, respectively. The OTUs of these module hubs were assigned to Proteobacteria (*Massilia*, *Pantoea*, and two unclassified genera), Firmicutes (unclassified), and Actinobacteriota (unclassified). Four nodes were classified as connectors in cultivars BL40, HR16, M15, and XD17, respectively. The OTUs of these connectors belonged to Proteobacteria (*Massilia*, *Pseudomonas*, *Methylobacterium*, and unclassified). No network hubs were found at all in the Z-P plot analyses of the phyllosphere. For the Z-P plot analyses of the endosphere, 99.86% of nodes were assigned to peripherals, while only one node was classified as a connector belonging to Proteobacteria (*Sphingomonas*), and no module hubs and network hubs were found at all.

### Identification of keystone taxa and functional profiling

Genera with a relative abundance > 1% were implemented in correlation analyses based on alpha and beta diversity of bacterial communities among different cultivars. The results indicated that various genera showed a significantly positive or negative correlation with alpha and beta diversity (*P* < 0.05) in the phyllosphere and endosphere based on Spearman correlation test (Table S6). Three genera were also shown to be part of the phyllosphere microbial networks, indicating their importance for intra-community interactions or dominance in the microbial ecosystem. The three genera were assigned to *Pantoea*, *Massilia*, and *Pseudomonas*. *Pantoea* showed a significantly negative correlation with alpha and beta diversity (*P* < 0.01), while *Massilia* and *Pseudomonas* showed a significantly positive correlation with alpha diversity (*P* < 0.01) in the phyllosphere (Table S6). However, these bacterial genera were not found in the endosphere microbial networks of the different wheat varieties. This observation indicates that *Pantoea*, *Massilia*, and *Pseudomonas* may be keystone species, and play an important role for microbial community regulation in the wheat phyllosphere.

The potential functions of microbial communities in the phyllosphere of different wheat cultivars were predicted via Tax4Fun analysis of the amplicon dataset (Table S7, Figure S3). In total, 348 KEGG homologs were identified, which were related to Metabolism, Genetic Information Processing, Human Diseases, Cellular Processes, and Environmental Information Processing. Among the identified functional pathways, most were associated with metabolic pathways. Compared to other pathways, Membrane Transport, Carbohydrate Metabolism, Amino Acid Metabolism, Signal Transduction, and Cellular Community (Prokaryotes) accounted for the highest percentage. It is worth noting that functions related to human diseases (i.e., Drug Resistance: Antimicrobial) accounted for the primary functional category. However, there was no significant difference in the 19 functional pathways (level 2).

### Correlations between bacterial communities and leaf length and width, and aphid hazard grade

Correlation analyses for leaf length, leaf width, and aphid hazard grade with alpha diversity of the bacterial communities were conducted with the Spearman correlation test (Table 2). The results indicated that the leaf length exhibited a significantly positive correlation with the alpha diversity indices Inv_Simpson (*P* = 0.007), Shannon (*P* = 0.005), Pielou_evenness (*P* = 0.004), and Chao1 (*P* < 0.001) in the wheat phyllosphere. However, no correlation was found between alpha diversity of endosphere-associated bacteria and leaf length, leaf width, and aphid hazard grade.

**Table 2:** Correlation of leaf length (LL), leaf width (LW), and aphid hazard grade (AG) with diversity indices and predominant bacteria (relative abundance > 1%) based on Spearman correlation test

Phyllosphere					Endosphere			
	LL	LW	AG			LL	LW	AG
Shannon	0.350^**^	0.097	0.333		Shannon	0.232	0.074	-0.381
Inv_Simpson	0.336^**^	0.033	0.333		Inv_Simpson	0.195	0.063	-0.310
Observed_richness	0.236	0.130	0.286		Observed_richness	0.199	-0.024	-0.286
Pielou_evenness	0.356^**^	0.094	0.476		Pielou_evenness	0.226	0.099	-0.357
Chao1	0.461^**^	0.152	0.286		Chao1	0.186	-0.028	-0.286
*Pantoea*	0.071	-0.148	0.452		*Pantoea*	0.029	-0.025	0.167
*Exiguobacterium*	-0.160	0.024	-0.881**		*Exiguobacterium*	-0.222	0.003	-0.476
*Massilia*	0.259^*^	0.091	0.429		*Curtobacterium*	0.078	0.041	-0.238
*Frigoribacterium*	0.435^**^	0.265^*^	0.548		*Frigoribacterium*	0.193	0.225	0.119
*Curtobacterium*	0.285^*^	0.342^**^	-0.286		*Erwinia*	0.107	0.045	0.095
*Erwinia*	0.041	-0.131	0.571		*Mycetocola*	0.205	0.217	-0.048
*Pseudomonas*	0.321^**^	0.110	0.214		*Serratia*	0.197	-0.145	0.381
*Sphingomonas*	0.217	0.133	0.524		*Sanguibacter*	0.055	0.117	-0.048
*Mycetocola*	0.340^**^	0.279^*^	-0.357		*Allorhizobium*	0.132	0.123	0.238
*Hymenobacter*	0.262^*^	0.188	0.000					
*Arthrobacter*	0.166	0.027	-0.357					

^*^Significant difference at the *P* = 0.05 level; ^**^Significant difference at *P* < 0.01 level

The correlation between the relative abundance of predominant bacteria in the wheat phyllosphere (genera with relative abundance > 1%) and the leaf length and width were further analyzed (Table 2). Leaf length showed a significant positive correlation with relative abundances of *Massilia* (*P* = 0.039), *Frigoribacterium* (*P* < 0.001), *Curtobacterium* (*P* = 0.023), *Pseudomonas* (*P* = 0.010), *Mycetocola* (*P* = 0.006), and *Hymenobacter* (*P* = 0.037). Leaf width exhibited a significantly positive correlation with the relative abundance of *Frigoribacterium* (*P* = 0.034), *Curtobacterium* (*P* = 0.006), and *Mycetocola* (*P* = 0.026).

A Mantel test was conducted and confirmed that aphid feeding affected leaf-associate microbial community structures in (Table S8); the aphid hazard grade (AG) was significantly correlated with phyllosphere-associated microbial communities in different wheat varieties (*P* < 0.05). The correlation between the relative abundance of predominant bacteria and the aphid hazard grade was further analyzed (Table 2). The results showed a significantly negative correlation between AG and the relative abundance of *Exiguobacterium* in the wheat phyllosphere (*P* = 0.004).

## Discussion

Microbes that colonize the rhizosphere, phyllosphere, and endosphere of plants can promote plant development and provide protection under abiotic and biotic stress [10, 12]. Plant-colonizing microbes are either transferred by air, wind, soil, water or vertically transmitted via seeds [42, 43]. With the advancement of sequencing technologies and development of molecular techniques in recent years,  many assemblages and functions of leaf-associated microbiomes were explored for various crop plants [27, 44–46]. In this study, the bacterial communities within the endosphere and phyllosphere of eight wheat varieties were explored via high-throughput sequencing of 16S rRNA gene fragments.

The extent to which host plant genotypes can shape their phyllosphere microbiome composition is still unclear. Previous research indicated that bacterial and fungal communities were significantly affected by the genotype in the lettuce phyllosphere [47], while the host genotype had a only a minor effect on the root-associated microbiome in *Arabidopsis thaliana* [11]. In the present study, alpha and beta diversity analyses together with dissimilarity tests showed that bacterial communities in the endosphere and phyllosphere of wheat plants showed significant differences among most cultivars. Differences in bacterial communities of the endosphere and phyllosphere may occur due to different permeability of distinct cultivars to the colonization of endophytic bacteria [27]. In this study, all eight wheat varieties were grown in the same field under the same environmental conditions to rule out external influences. Therefore, the results indicate that the host genotype has pronounced effects on plant-associated bacterial communities in wheat.

Previous studies have shown that the predominant members of leaf-associate microbial communities are bacteria and colonize them in high densities [48]; mainly members of the phyla Actinobacteria, Proteobacteria, and Bacteroidetes [10, 46]. The current study found that the average relative abundance of Proteobacteria was lower in the endosphere than in the phyllosphere among the eight wheat cultivars. The genus *Pantoea* was prevalent therein. Members of the genus *Pantoea* were often found on leaves of various ceral crops, including rice and maize [27, 49]. The genera *Pantoea*, *Exiguobacterium*, *Frigoribacterium*, *Curtobacterium*, and *Erwinia* were shared between the bacterial communities in the endosphere and phyllosphere of the eight analyzed wheat cultivars. These genera were often reported as core microorganisms in plant leaves. For instance, *Exiguobacterium*, *Erwinia*, and *Pantoea* were the most consistently found across samples in lettuce [50], and the genus *Curtobacterium* was identified as core member in sugarcane [51].

Network analysis is commonly used to infer interactions among microbial species, such as mutualism and competition [35, 52]. Understanding interactions among microbial can reveal critical factors that affect complex microbial community structures across spatial gradients [53]. In this study, networks were constructed by analyzing compositional data using a Spearman correlation metric. Although researchers point out that correlations based on this method may lead to spurious associations based on compositional data [54, 55], it is still widely used and can provide valuable information [36, 37]. In our samples, the number of total links, nodes, and modules was significantly higher in the phyllosphere than in the endosphere in most wheat cultivars, indicating that the phyllosphere hosted more complex and stable networks, with closer and better-connected nodes. High network complexity is an indicator for stable communities [56]. Moreover, we found that 88.50-99.54% and 64.95–93.51% of the interactions within modules were positive in the phyllosphere and endosphere, respectively, suggesting that interactions among different bacterial species were mutualistic or neutral. This may be more advantageous to form a stable microbial community structure.

The host genotype directly affects distinct microorganisms, which then may interact with other members of the microbiota to influence the composition and diversity of the community as a whole [57]. Our results suggest that the genera *Pantoea*, *Massilia*, and *Pseudomonas* were embedded in complex intra-community interaction networks, and showed a strong correlation (positive or negative) with alpha or beta diversity of the bacterial community in the phyllosphere. *Pantoea* spp. can produce phytohormones that promote plant growth and suppress *Botrytis cinerea* in tomato leaves [58]. The genus *Massilia* was reported in the leaf microbiomes of spinach [59], rice [27], and lettuce [50], and identified as a main component of agricultural aerosols in central California [60]. Different *Pseudomonas* spp. strains isolated from wheat leaves had antagonistic effects on the fungal pathogens *Fusarium* and *Alternaria* [26]. *Pseudomonas* *protegens* CS1 isolated from the phyllosphere of lemon can produce the siderophore pyochelin as well as reactive oxygen species and has a strong inhibition activity towards *Xanthomonas citri* subsp. *Citri* [61]. These findings suggest that these three genera play a role in shaping the phyllosphere microbial community in wheat and contribute beneficial functions to holobiont functioning. Additionally, we also observed similar ecological functions of microbial communities in the phyllosphere of eight wheat cultivars. A recent study showed that regardless of ecosystem or spatial and environmental heterogeneity, there is a robust regional core phyllosphere community that maintains the structural and functional stability of the microbial community [62]. These findings are concordant with the importance of the core community in driving energy and nutrient metabolism in the phyllosphere.

The leaf traits of different plant species vary, resulting in substantial differences in their complex microbial communities in the phyllosphere [63]. For instance, the relative abundance of the genera *Microvirga*, *Nocardioides*, and *Gaiella* was significantly negatively correlated with leaf length, but was significantly positively correlated with the alpha and beta diversity of bacterial communities in the endosphere of different rice cultivars [27]. In the present study, we also found that the relative abundance of specific bacterial species showed a significantly positive correlation with leaf length and width, respectively, and that leaf length showed a significant positive correlation with the alpha diversity of bacterial communities in the wheat phyllosphere. Among them, *Massilia* and *Pseudomonas* were also part of the phyllosphere microbial network. These findings suggest phenotypic adaptions of the host in response to these taxa. Further research will be required to decipher the underlying mechanisms and to identify potential effects on the overall host performance as a response to these microbes.

Pests cause substantial damage to wheat production, especially aphids. At present, the aphids *S*. *avenae* and *R*. *padi* are the main pests that affect production of wheat in China [23, 24]. Factors that affect herbivores’ feeding on the host may also influence the colonization and growth of leaf-associated microorganisms [5]. A previous study demonstrated that herbivore damage from *Scaptomyza nigrita* reshapes the native leaf microbiome in bittercress [5]. Our study indicated that aphid AG significantly correlated with phyllosphere-associated microbial communities in different wheat varieties. Additionally, we found for the first time that the number of detected aphids was significantly negatively correlated with the relative abundance of the genus *Exiguobacterium* in different wheat varieties. These results suggest that the feeding of aphids may have caused changes in phyllosphere-associated microbial communities, or that members of the genus *Exiguobacterium* may have an adverse effect on wheat aphids.

*Exiguobacterium* is widely distributed in different environments, such as seawater, soils, sediments, glaciers, and permafrost [64]. It has various unique properties, including halophilic or alkalophilic and thermophilic or psychrophilic growth preferences, and can decompose complex organic pollutants, transform heavy metals, and promote plant growth [64]. Some *Exiguobacterium* spp. strains that promote plant growth have one or more traits that are beneficial to plants, such as the production of acetic acid, siderophores and hydrogen cyanide, and phosphate solubilization properties, and antagonistic effects against various plant pathogens [64]. *Exiguobacterium acetylicum* 1 P, isolated from rhizosphere soil in *Malus domestica*, has various plant growth-promoting properties, which positively affected the nutrient absorption and growth parameters of greenhouse wheat seedlings [65]. In addition, *Exiguobacterium* sp. EM9 exhibited high antagonistic activity against plant pathogens, improving the emergence rate, root length, and plant dry weight of capsicum and tomato following seed treatments [66]. Overall, our findings indicate that the microbial community structure in the phyllosphere may be affected by aphids feeding on wheat leaves. Whether specific members of the genus *Exiguobacterium* have insecticidal activity against aphids needs further verification.

## Conclusions

In conclusion, our findings revealed that leaf-associated bacterial communities in wheat were mainly driven by the host genotype. *Pantoea*, *Exiguobacterium*, *Frigoribacterium*, *Curtobacterium*, and *Erwinia* were identified as predominant genera and were shared between the phyllosphere and endosphere. Moreover, the phyllosphere hosted more complex and stable microbial networks than the endosphere in most wheat cultivars. The genera *Pantoea*, *Massilia*, and *Pseudomonas* were found to play a key role in shaping the bacterial community in the phyllosphere of wheat. Additionally, wheat plants showed specific phenotypic adaptations to the genera *Massilia* and *Pseudomonas*. The phyllosphere-associated microbial community structure correlated with the number of aphids feeding on wheat leaves. The abundance of the genus *Exiguobacterium* was significantly negatively correlated with the aphid hazard grade. An in-depth study of antagonistic effects and mechanisms of leaf-associated microorganisms on pests could provide new solutions for sustainable crop production and integrated pest control. Further research should focus on species-level identifications of leaf-associated beneficial microorganisms, determination of their antagonistic effects and mechanisms on pests, as well as additional beneficial roles in wheat and potentially other crops by implementing *in vitro* and field studies.

### Electronic supplementary material

Below is the link to the electronic supplementary material.

Supplementary Material 1

## Data Availability

The sequencing data is available at the NCBI Sequence Read Archive under accession no. PRJNA923742.
