# Supplementary material for: Exploration of phyllosphere microbiomes in wheat varieties with differing aphid resistance
Source: Environ Microbiome. 2023 Oct 24;18:78. doi: 10.1186/s40793-023-00534-5 (PMC10594911; doi:10.1186/s40793-023-00534-5)
Supplement: Supplementary file 1 — Supplementary Material 1 [file 40793_2023_534_MOESM1_ESM.docx]

Table S1 Wheat cultivars implemented in the present study

| Code | Cultivar | Source |
| --- | --- | --- |
| HR16 | Heng-r16-5152 | Hebei Academy of Agricultural and Forestry Sciences, China |
| L112 | Luo-11238-147-41 | Luoyang Academy of Agriculture and Forestry Sciences, China |
| L01 | Lan-01-368 | Gansu Academy of Agricultural Sciences, China |
| H05 | Han-05-5093 | Handan Academy of Agricultural Sciences, China |
| BL40 | BL4071 | Mianyang Academy of Agricultural Sciences, China |
| K13 | Ke-13-487 | Heilongjiang Academy of Agricultural Sciences, China |
| M15 | Mian-15Z30 | Mianyang Academy of Agricultural Sciences, China |
| XD17 | Xindong-17 | Xinjiang Academy of Agricultural Sciences, China |

Table S2 Dissimilarity tests of bacterial communities in the phyllosphere of different wheat varieties based on analysis of similarities with Adonis (PERMANOVA)

| PERMANOVA | XD17 | L112 | K13 | HR16 | L01 | BL40 | M15 | H05 |
| --- | --- | --- | --- | --- | --- | --- | --- | --- |
| XD17 |  | 0.001 | 0.001 | 0.004 | 0.008 | 0.001 | 0.001 | 0.004 |
| L112 | 23.24 |  | 0.001 | 0.001 | 0.001 | 0.001 | 0.001 | 0.001 |
| K13 | 4.3153 | 8.7372 |  | 0.026 | 0.230 | 0.293 | 0.006 | 0.037 |
| HR16 | 5.7458 | 15.069 | 2.1623 |  | 0.008 | 0.014 | 0.001 | 0.004 |
| L01 | 2.873 | 9.4208 | 1.2744 | 2.6175 |  | 0.030 | 0.001 | 0.453 |
| BL40 | 6.414 | 8.2263 | 1.2065 | 2.8389 | 2.3408 |  | 0.028 | 0.076 |
| M15 | 14.43 | 13.374 | 3.542 | 3.9204 | 4.8838 | 2.4861 |  | 0.004 |
| H05 | 4.2802 | 5.4801 | 2.0127 | 3.0358 | 0.9712 | 2.0148 | 3.3299 |  |

The values in the upper triangular matrices are significance values (*p*-value). The values of the lower triangular matrices for MRPP are F values.

Table S3 Dissimilarity tests of bacterial communities in the endosphere of different wheat varieties based on analysis of similarities with Adonis (PERMANOVA)

| PERMANOVA | XD17 | L112 | K13 | HR16 | L01 | BL40 | M15 | H05 |
| --- | --- | --- | --- | --- | --- | --- | --- | --- |
| XD17 |  | 0.004 | 0.430 | 0.013 | 0.006 | 0.066 | 0.258 | 0.010 |
| L112 | 5.7557 |  | 0.007 | 0.001 | 0.001 | 0.001 | 0.001 | 0.002 |
| K13 | 0.9761 | 4.174 |  | 0.004 | 0.006 | 0.045 | 0.007 | 0.049 |
| HR16 | 2.164 | 7.9922 | 2.6685 |  | 0.025 | 0.046 | 0.001 | 0.001 |
| L01 | 2.6992 | 8.8661 | 2.2656 | 2.0849 |  | 0.036 | 0.002 | 0.004 |
| BL40 | 1.9684 | 9.2633 | 2.2679 | 1.9366 | 2.0156 |  | 0.114 | 0.028 |
| M15 | 1.262 | 10.0661 | 3.0468 | 2.6577 | 3.322 | 1.6191 |  | 0.001 |
| H05 | 2.4894 | 6.2358 | 1.9089 | 3.0739 | 2.6616 | 2.4281 | 3.4297 |  |

The values in the upper triangular matrices are significance values (*p*-value). The values in the lower triangular matrices for MRPP are F values.

Table S4 Topological properties of the empirical molecular ecological networks (MENs) and their associated random MENs.

|  | Cultivar/ Network Indices | Empirical Network Indices | | | | | | | |  | Random Network Indices | | |
| --- | --- | --- | --- | --- | --- | --- | --- | --- | --- | --- | --- | --- | --- |
|  |  | Similarity  threshold | Total nodes | Total links | R^2^ of power-law | Average degree (avgK) | Average clustering coefficient (avgCC) | Average path distance (GD) | Modularity (the number of modules) |  | Average clustering coefficient (avgCC) | Average path distance (GD) | Modularity |
| Phyllosphere | HR16 | 0.91 | 174 | 219 | 0.917 | 2.517 | 0.197 | 7.261 | 0.783 (31) |  | 0.014 ± 0.008 | 4.962 ± 0.175 | 0.670 ± 0.011 |
|  | L112 | 0.91 | 110 | 113 | 0.892 | 2.055 | 0.234 | 3.436 | 0.880 (25) |  | 0.015 ± 0.012 | 6.351 ± 0.657 | 0.753 ± 0.015 |
|  | L01 | 0.91 | 139 | 159 | 0.759 | 2.288 | 0.210 | 7.910 | 0.848 (28) |  | 0.013 ± 0.008 | 5.674 ± 0.263 | 0.712 ± 0.013 |
|  | H05 | 0.91 | 122 | 119 | 0.824 | 1.951 | 0.228 | 3.238 | 0.894 (28) |  | 0.014 ± 0.009 | 7.245 ± 0.850 | 0.794 ± 0.015 |
|  | BL40 | 0.91 | 201 | 296 | 0.820 | 2.945 | 0.316 | 6.820 | 0.837 (29) |  | 0.015 ± 0.008 | 4.621 ± 0.109 | 0.607 ± 0.010 |
|  | K13 | 0.91 | 153 | 203 | 0.831 | 2.654 | 0.276 | 4.837 | 0.832 (24) |  | 0.014 ± 0.009 | 4.831 ± 0.136 | 0.641 ± 0.011 |
|  | M15 | 0.91 | 205 | 319 | 0.893 | 3.112 | 0.240 | 4.243 | 0.774 (36) |  | 0.019 ± 0.007 | 4.219 ± 0.100 | 0.575 ± 0.009 |
|  | XD17 | 0.91 | 138 | 218 | 0.902 | 3.159 | 0.277 | 7.426 | 0.779(22) |  | 0.023 ± 0.008 | 4.133 ± 0.105 | 0.557 ± 0.012 |
| Endosphere | HR16 | 0.83 | 68 | 97 | 0.847 | 2.853 | 0.243 | 3.987 | 0.609 (11) |  | 0.056 ± 0.022 | 3.662 ± 0.174 | 0.538 ± 0.015 |
|  | L112 | 0.83 | 42 | 41 | 0.667 | 1.952 | 0.279 | 2.073 | 0.832 (10) |  | 0.036 ± 0.021 | 4.992 ± 0.870 | 0.683 ± 0.026 |
|  | L01 | 0.83 | 74 | 126 | 0.689 | 3.405 | 0.280 | 4.188 | 0.660 (10) |  | 0.047 ± 0.016 | 3.487 ± 0.102 | 0.486 ± 0.017 |
|  | H05 | 0.83 | 86 | 154 | 0.738 | 3.581 | 0.294 | 3.429 | 0.617 (14) |  | 0.080 ± 0.020 | 3.349 ± 0.124 | 0.458 ± 0.012 |
|  | BL40 | 0.83 | 70 | 93 | 0.843 | 2.657 | 0.184 | 3.954 | 0.742 (10) |  | 0.027 ± 0.017 | 4.261 ± 0.191 | 0.587 ± 0.018 |
|  | K13 | 0.83 | 68 | 102 | 0.789 | 3.000 | 0.217 | 2.863 | 0.585 (15) |  | 0.058 ± 0.019 | 3.386 ± 0.113 | 0.508 ± 0.016 |
|  | M15 | 0.83 | 65 | 67 | 0.673 | 2.062 | 0.188 | 4.480 | 0.788 (16) |  | 0.023 ± 0.012 | 5.592 ± 0.605 | 0.701 ± 0.016 |
|  | XD17 | 0.83 | 46 | 64 | 0.545 | 2.783 | 0.182 | 2.041 | 0.507 (11) |  | 0.087 ± 0.027 | 3.156 ± 0.192 | 0.477 ± 0.017 |

Table S5 Relationships between links in microbial interaction networks

|  | Cultivar | Total Links | Positive Links | Negative Links | Positive Interactions (%) | Negative Interactions (%) |
| --- | --- | --- | --- | --- | --- | --- |
| Phyllosphere | BL40 | 296 | 262 | 34 | 88.51 | 11.49 |
|  | H05 | 119 | 109 | 10 | 91.60 | 8.40 |
|  | HR16 | 219 | 206 | 13 | 94.06 | 5.94 |
|  | K13 | 203 | 172 | 31 | 84.73 | 15.27 |
|  | L01 | 159 | 158 | 1 | 99.37 | 0.63 |
|  | L112 | 113 | 100 | 13 | 88.50 | 11.50 |
|  | M15 | 319 | 292 | 27 | 91.54 | 8.46 |
|  | XD17 | 218 | 217 | 1 | 99.54 | 0.46 |
| Endosphere | BL40 | 93 | 77 | 16 | 82.80 | 17.20 |
|  | H05 | 154 | 144 | 10 | 93.51 | 6.49 |
|  | HR16 | 97 | 63 | 34 | 64.95 | 35.05 |
|  | K13 | 102 | 92 | 10 | 90.20 | 9.80 |
|  | L01 | 126 | 98 | 28 | 77.78 | 22.22 |
|  | L112 | 41 | 36 | 5 | 87.80 | 12.20 |
|  | M15 | 67 | 48 | 19 | 71.64 | 28.36 |
|  | XD17 | 64 | 58 | 6 | 90.63 | 9.38 |

Table S6 Correlation between predominant bacteria and α-diversity and β-diversity for bacterial communities in different cultivars based on Spearman correlation test.

|  | Species/Index | α-diversity | | | | |  | β-diversity |
| --- | --- | --- | --- | --- | --- | --- | --- | --- |
|  |  | Shannon | Inv_Simpson | Observed_richness | Pielou_evenness | chao |  | PCoA |
| Phyllosphere | *Pantoea* | -0.408^**^ | -0.396^**^ | -0.334^**^ | -0.422^**^ | -0.311^*^ |  | -0.845^**^ |
|  | *Exiguobacterium* | -0.037 | -0.084 | 0.063 | -0.048 | 0.007 |  | 0.760^**^ |
|  | *Massilia* | 0.733^**^ | 0.692^**^ | 0.712^**^ | 0.702^**^ | 0.620^**^ |  | -0.051 |
|  | *Frigoribacterium* | 0.669^**^ | 0.620^**^ | 0.555^**^ | 0.663^**^ | 0.576^**^ |  | 0.000 |
|  | *Curtobacterium* | 0.518^**^ | 0.477^**^ | 0.366^**^ | 0.523^**^ | 0.359^**^ |  | 0.289^*^ |
|  | *Erwinia* | 0.252^*^ | 0.282^*^ | 0.070 | 0.280^*^ | -0.119 |  | -0.279^*^ |
|  | *Pseudomonas* | 0.480^**^ | 0.422^**^ | 0.535^**^ | 0.457^**^ | 0.515^**^ |  | -0.188 |
|  | *Sphingomonas* | 0.617^**^ | 0.576^**^ | 0.485^**^ | 0.597^**^ | 0.440^**^ |  | -0.018 |
|  | *Mycetocola* | 0.707^**^ | 0.626^**^ | 0.640^**^ | 0.683^**^ | 0.572^**^ |  | 0.194 |
|  | *Hymenobacter* | 0.739^**^ | 0.706^**^ | 0.622^**^ | 0.722^**^ | 0.485^**^ |  | 0.021 |
|  | *Arthrobacter* | 0.366^**^ | 0.282^*^ | 0.384^**^ | 0.343^**^ | 0.607^**^ |  | 0.325^**^ |
| Endosphere | *Pantoea* | -0.121 | 0.011 | -0.312^*^ | -0.066 | -0.305^*^ |  | -0.369^**^ |
|  | *Exiguobacterium* | -0.358^**^ | -0.337^**^ | -0.287^*^ | -0.338^**^ | -0.277^*^ |  | 0.840^**^ |
|  | *Curtobacterium* | 0.436^**^ | 0.316^*^ | 0.462^**^ | 0.361^**^ | 0.343^**^ |  | -0.386^**^ |
|  | *Frigoribacterium* | 0.446^**^ | 0.380^**^ | 0.322^**^ | 0.440^**^ | 0.227 |  | -0.451^**^ |
|  | *Erwinia* | -0.096 | -0.046 | -0.206 | -0.053 | -0.095 |  | 0.024 |
|  | *Mycetocola* | 0.444^**^ | 0.369^**^ | 0.332^**^ | 0.409^**^ | 0.235 |  | -0.378^**^ |
|  | *Serratia* | 0.382^**^ | 0.354^**^ | 0.390^**^ | 0.353^**^ | 0.424^**^ |  | -0.056 |
|  | *Sanguibacter* | 0.277^*^ | 0.199 | 0.229 | 0.270^*^ | 0.202 |  | -0.025 |

^*^Significant difference at the *P* = 0.05 level; ^**^Significant difference at the *P* = 0.01 level.

Table S7 Relative abundance of potential functional pathways in the phyllosphere of different wheat cultivars

| Level 3 | Level 2 | HR16 | L112 | L01 | H05 | BL40 | K13 | M15 | XD17 |
| --- | --- | --- | --- | --- | --- | --- | --- | --- | --- |
| Metabolism | Global and overview maps | 36.3 | 35.7 | 35.9 | 35.6 | 35.7 | 35.6 | 36.5 | 35.3 |
| Metabolism | Carbohydrate metabolism | 10.4 | 11.2 | 10.4 | 10.6 | 10.5 | 10.1 | 10.2 | 10.4 |
| Metabolism | Amino acid metabolism | 6.8 | 7.1 | 6.6 | 6.6 | 6.7 | 6.7 | 6.8 | 6.4 |
| Metabolism | Metabolism of cofactors and vitamins | 3.4 | 3.3 | 3.4 | 3.4 | 3.4 | 3.4 | 3.5 | 3.4 |
| Metabolism | Energy metabolism | 3.2 | 3.1 | 3.2 | 3.2 | 3.2 | 3.2 | 3.1 | 3.3 |
| Metabolism | Lipid metabolism | 3.2 | 2.9 | 2.9 | 2.7 | 2.9 | 2.9 | 3.1 | 2.7 |
| Metabolism | Nucleotide metabolism | 2.1 | 2.4 | 2.2 | 2.3 | 2.2 | 2.2 | 2.2 | 2.2 |
| Metabolism | Xenobiotics biodegradation and metabolism | 2.4 | 2.2 | 2.3 | 2.2 | 2.3 | 2.3 | 2.4 | 2.2 |
| Metabolism | Metabolism of other amino acids | 1.5 | 1.4 | 1.5 | 1.5 | 1.5 | 1.5 | 1.5 | 1.5 |
| Metabolism | Biosynthesis of other secondary metabolites | 1.6 | 1.2 | 1.5 | 1.4 | 1.5 | 1.5 | 1.7 | 1.4 |
| Metabolism | Metabolism of terpenoids and polyketides | 1.4 | 1.2 | 1.2 | 1.2 | 1.3 | 1.4 | 1.4 | 1.1 |
| Human Diseases | Drug resistance: Antimicrobial | 1.3 | 1.1 | 1.4 | 1.4 | 1.3 | 1.3 | 1.2 | 1.5 |
| Genetic Information Processing | Replication and repair | 1.3 | 1.4 | 1.4 | 1.4 | 1.4 | 1.3 | 1.4 | 1.3 |
| Genetic Information Processing | Translation | 1.2 | 1.5 | 1.3 | 1.3 | 1.3 | 1.2 | 1.3 | 1.2 |
| Genetic Information Processing | Folding, sorting and degradation | 0.9 | 1.0 | 0.9 | 0.9 | 0.9 | 0.9 | 0.9 | 0.9 |
| Environmental Information Processing | Membrane transport | 7.5 | 8.0 | 8.5 | 8.6 | 7.7 | 7.9 | 7.1 | 9.0 |
| Environmental Information Processing | Signal transduction | 4.3 | 4.6 | 4.3 | 4.5 | 4.7 | 4.8 | 4.5 | 4.4 |
| Cellular Processes | Cellular community - prokaryotes | 4.9 | 4.7 | 5.0 | 5.0 | 5.1 | 5.2 | 4.9 | 5.3 |
| Cellular Processes | Cell motility | 1.4 | 1.4 | 1.6 | 1.6 | 1.5 | 1.6 | 1.4 | 1.7 |

Table S8 Mantel test for correlations between bacterial community structures and aphid hazard grade (AG) based on Bray-Curtis and Jaccard distances

| Envs.factor  Aphid hazard grade (AG) | Bray-Curtis | |  | Jaccard | |
| --- | --- | --- | --- | --- | --- |
|  | R^2^ | *P* |  | R^2^ | *P* |
| Phyllosphere | 0.12 | 0.03 |  | 0.14 | 0.04 |
| Endosphere | -0.01 | 0.58 |  | 0.03 | 0.29 |


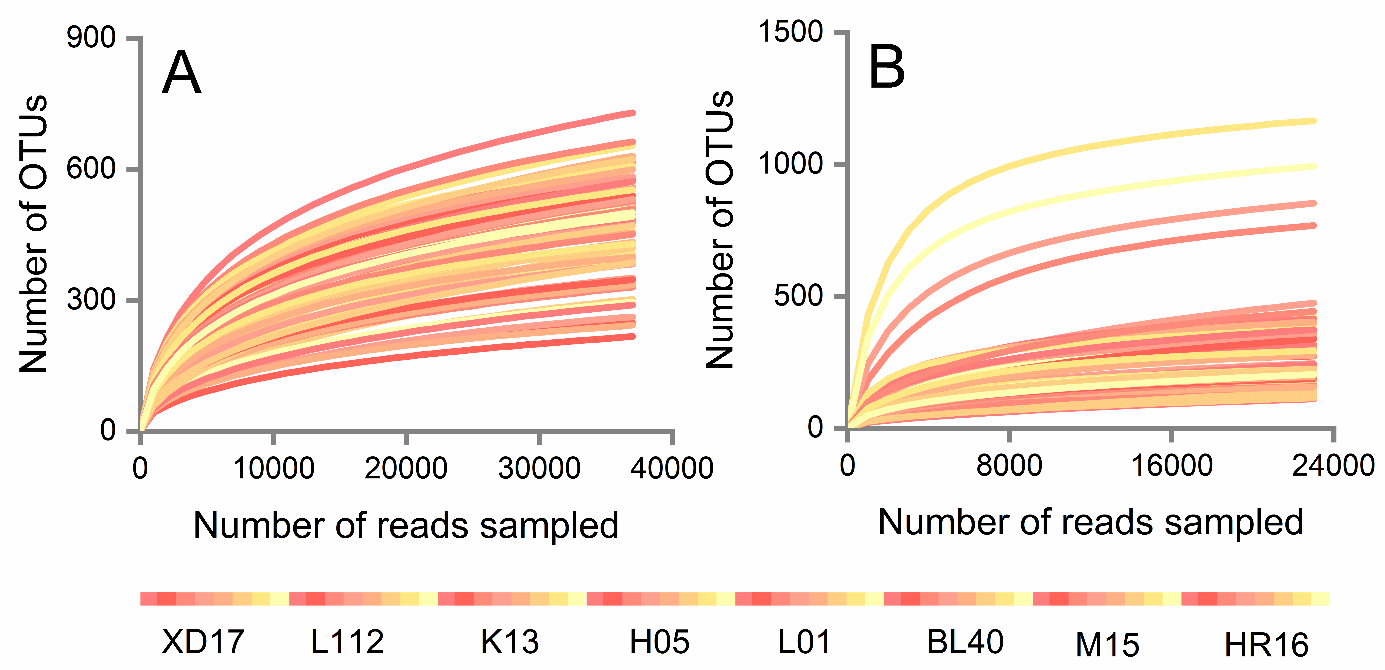
 Figure S1 Rarefaction curves for wheat phyllosphere (A) and endosphere (B) bacterial communities in this study with clustering at 97% sequence similarity.


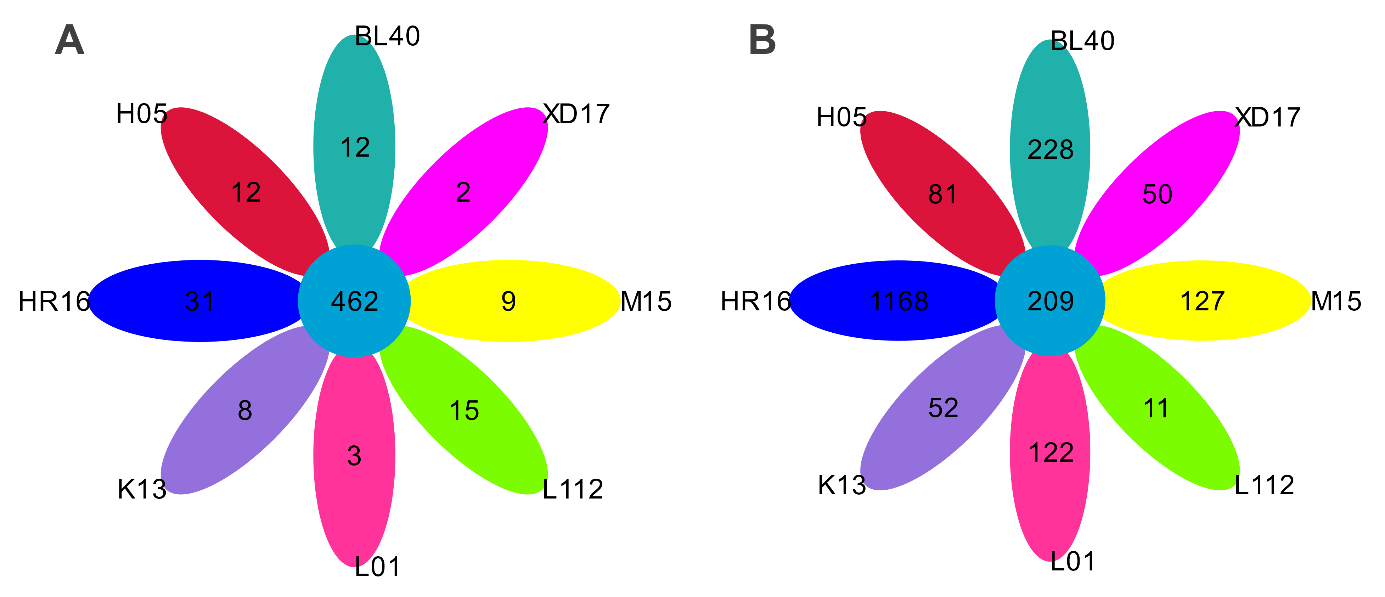


Figure S2 Venn diagrams showing the numbers of shared OTUs in the phyllosphere (A) and endosphere (B) of different wheat cultivars.


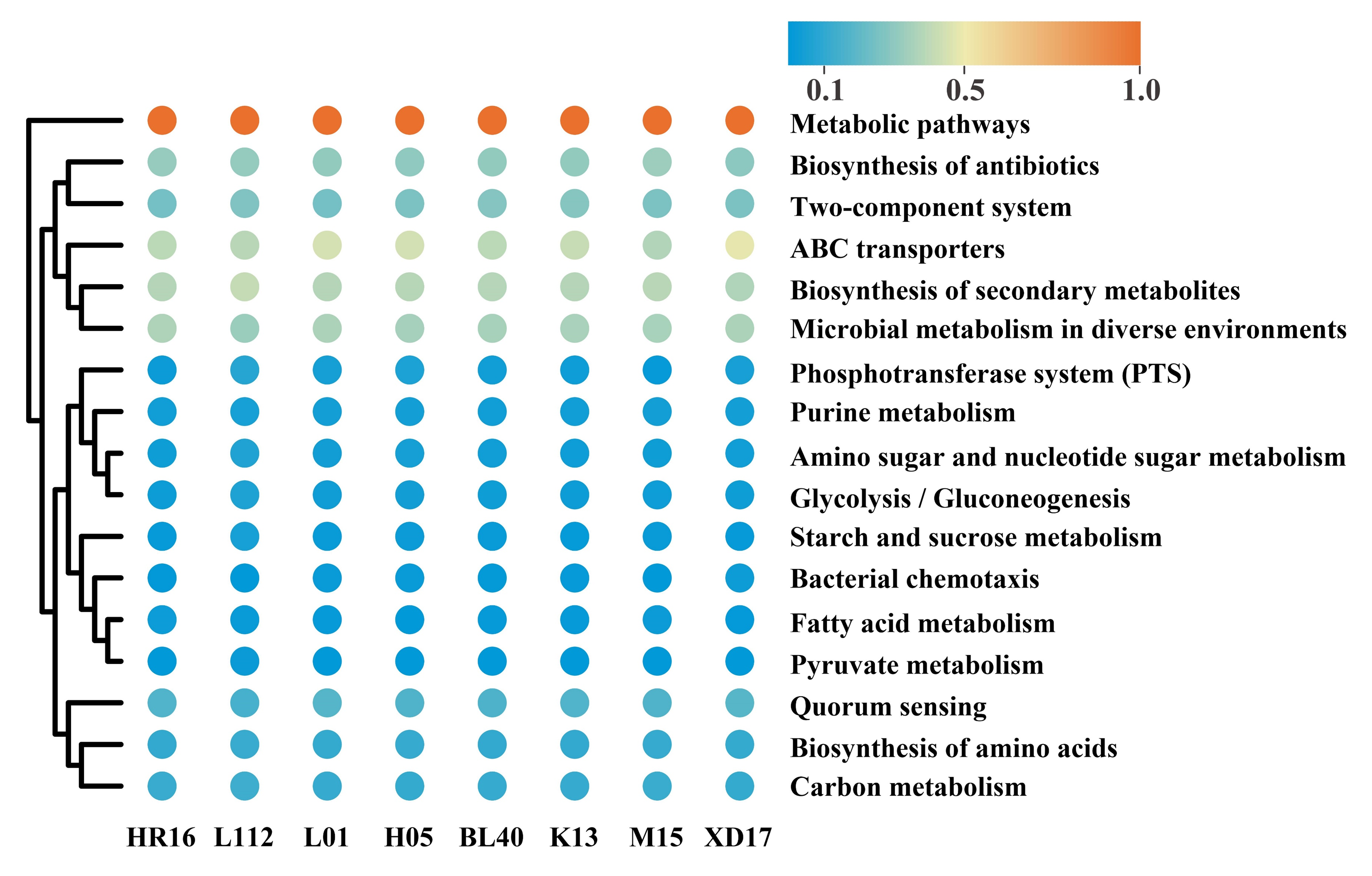


Figure S3 Heatmap of the functional predictions for bacterial communities in the phyllosphere of different wheat varieties.
